# Increased Expression of lncRNA UCA1 and HULC Is Required for Pro-inflammatory Response During LPS Induced Sepsis in Endothelial Cells

**DOI:** 10.3389/fphys.2019.00608

**Published:** 2019-05-21

**Authors:** Ying Chen, Yao Fu, Yan-fei Song, Nan Li

**Affiliations:** Department of Intensive Care Unit, The First Hospital of Jilin University, Changchun, China

**Keywords:** long non-coding RNA, lncRNA, HULC, UCA1, MALAT-1, sepsis, LPS, endothelial cells

## Abstract

Systemic uncontrolled inflammatory response, also termed as sepsis, is responsible for many mortalities. Bacterial endotoxin, lipopolysaccharide (LPS), is a major cause of sepsis in endothelial cells. Even though a lot of research has been done to define underlying mechanisms of LPS induced sepsis, the role of long non-coding RNAs (lncRNAs), a group of >200 kb RNAs in sepsis is not well-defined. Expression of pro-inflammatory mediators *IL6*, *ICAM1*, and *VCAM1* (which encodes interleukin-6, intercellular adhesion molecule-1, and vascular cell adhesion molecule-1, respectively) were determined following LPS treatment of human dermal microvascular endothelial cells (HMECs) for 24 h to confirm sepsis induction. RNA immunoprecipitation (RIP) analysis was performed using the chromatin modifying proteins (CMPs), heterogeneous nuclear ribonucleoprotein (hnRNP) K and corepressors of the RE-1 silencing transcription factor (coREST) as individual baits. Quantitative real time polymerase chain reaction (qRT-PCR) was performed on RNA isolated from immunoprecipitated pellets for six different lncRNAs. The effect of the differentially expressed lncRNAs were determined by ectopic overexpression of the lncRNAs before induction of sepsis. Expression of *IL6*, *ICAM1*, and *VCAM1* were significantly upregulated following treatment of the HMECs with LPS for 24 h confirming induction of sepsis. RIP and qRT-PCR analysis revealed that the lncRNAs HULC, UCA1, and MALAT-1 were significantly enriched with the CMPs after sepsis. RNA interference using siRNAs targeting HULC and UCA1, but not MALAT-1, decreased the expression of *IL6*, *ICAM1*, and *VCAM1* to endogenous levels. Our results were further validated in an *in vivo* model of sub-lethal LPS-induced sepsis, whereby siRNA mediated knockdown of UCA1 and HULC lncRNAs prevented induction of VCAM1, ICAM1, and IL6, as assayed by immunohistochemistry. Cumulatively, these results suggest that LPS induced *in vitro* sepsis in endothelial cells and induction of pre-inflammatory mediators are at least in part due to increased expression of the UCA1 and HULC lncRNAs.

## Introduction

Sepsis is an uncontrolled systemic inflammatory response that can affect multiple organs in the body and is a major cause of worldwide mortality (Dauphinee and Karsan, 2006; Vincent et al., 2011; Randolph and McCulloh, 2014). When lipopolysaccharide (LPS), present within the cell wall of Gram-negative bacteria, encounters endothelial cells, a signaling cascade is induced resulting in sepsis (Dauphinee and Karsan, 2006) in initiated.

Endothelial cells line the microcirculatory route, maintaining an antithrombotic surface and in turn regulating vasomotor tonicity and normal blood flow (Danese et al., 2007; Chelazzi et al., 2015). Exposure of the microvascular endothelium to inflammatory cytokines or to endotoxins like LPS result in induced expression of pro-inflammatory markers inclusive of interleukin-6 (IL-6), intercellular adhesion molecule-1 (ICAM-1), and vascular cell adhesion molecule-1 (VCAM-1) (Danese et al., 2007; Chelazzi et al., 2015).

It is well documented that abnormal expression of both small (<200 kb) and long (lncRNA; >200 kb) non-coding RNAs (ncRNAs) is associated with different human diseases (Bussemakers et al., 1999; Srikantan et al., 2000; Iacoangeli et al., 2004; Petrovics et al., 2004; Gupta et al., 2010). In fact, lncRNA expression also seems to be involved in normal development processes as is evidenced by impaired neuronal development by knockdown of either *Pantr2* (linc-Brn1b) or *Evf2* (Bond et al., 2009; Sauvageau et al., 2013). Given the central role of LPS in mediating sepsis and septic shock it is not surprising that the mechanisms regulating the pathophysiology of sepsis has been studied in detail (Beck et al., 2006; Singh et al., 2016; Yu et al., 2017).

However, beyond a few studies (Singh et al., 2016; Chowdhury et al., 2017), including one which showed that the lncRNAs EGO (NONHSAT087634) and HOTAIRM1 (NONHSAT119666) are alternatively spliced and that expression of lnc-IL7R is increased during LPS induced sepsis (Chowdhury et al., 2017), not much is known about how altered lncRNA expression is related to pathophysiology of sepsis. HULC (highly upregulated in liver cancer) is a 16 kb lncRNA at chromosomal location 6p24.3 and is has been shown to be overexpressed in patients with liver cancer (Ruan et al., 2018). LncRNA urothelial carcinoma-associated 1 (UCA1) at chromosomal location 19p13.12 was initially identified in human bladder carcinoma but plays widespread role in inflammation and tumorigenesis (Liu et al., 2019). It has been shown to regulate Hippo signaling pathway as well as miRNA sponge function of miR-96 and miR-135a (Liu et al., 2019). Hence, our goal was to identify putative role of lncRNA in LPS-induced sepsis.

The lncRNAs directly interact with chromatin modifying proteins (CMPs) – heterogeneous nuclear ribonucleoprotein K (hnRNP K), corepressors of the RE-1 silencing transcription factor (coREST), polycomb repressor complex 2, and Sin3A. This interaction results in lncRNA-mediated changes in lineage specific gene expression and epigenetic silencing (Nagano et al., 2008; Huarte et al., 2010; Nagano and Fraser, 2011; Pandey and Kanduri, 2011). Therefore, RNA immunoprecipitation (RIP) analysis using CMP proteins as bait affords a way of elucidating lncRNA involved in a particular physiological context. Using hnRNP K and coREST as the baits, RIP analysis revealed that expression of three lncRNAs, whose expression is significantly upregulated during LPS-induced sepsis in endothelial cells *in vitro*, mediates the inflammatory response.

## Materials and Methods

### Cell Culture and LPS Treatment

Human dermal microvascular endothelial cells (HMECs; Lonza, Basel, Switzerland) were cultured as described previously (Rydkina et al., 2010). Briefly, cells were cultured in MCDB 131 medium (Caisson’s Laboratories) supplemented with 10% fetal bovine serum (Thermo Fisher Scientific, Shanghai, China), 10 ng/ml epidermal growth factor (Thermo Fisher Scientific), 1 μg/ml hydrocortisone (Sigma-Aldrich, Shanghai, China), and 10 mM L-Glutamine (Thermo Fisher Scientific). LPS from *Escherichia coli* (*E. coli* O111: B4; Invivogen, San Diego, CA, United States) was resuspended in distilled water just before use. HMECs were treated with 1 μg/ml LPS for 24 h.

### RNA Immunoprecipitation

After 24 h, untreated and LPS treated HMECs were washed twice with ice-cold PBS and then lysed in buffer containing 1.28 M sucrose, 40 mM Tris–HCl (pH 7.5), 20 mM MgCl_2_, and 4% (v/v) Triton X-100. Followed by centrifugation at 2700 ×*g* for 10 min. The nuclear pellet was resuspended in RIP buffer (Thermo Fisher Scientific). The hnRNP K antibody (EP943Y clone, ab52600; Abcam, Waltham, MA, United States) and coREST antibody (polyclonal ChIP grade; 07–455; Millipore, United States) was used to perform immunoprecipitation using magnetic agarose A/G beads (Thermo Fisher Scientific) following manufacturer’s protocol. Control RIP using IgG was performed. Immunoprecipitates were washed thrice with RIP buffer before being processed as described in the next section.

### RNA Isolation From Immunoprecipitates and qRT-PCR

RNA from the immunoprecipitated pellets or from total cell lysates were isolated using the RNeasy kit (Qiagen, Grand Island, NY, United States). The KAPA SYBR^®^ FAST One-Step qRT-PCR Kit (KAPA Biosystems, Wilmington, MA, United States) was used using 1 μg of RNA as input. Primers used for amplification were:

**Table:** 

*ICAM1*	Forward – 5′- GGCCTCAGTCAGTGTGA -3′ Reverse – 5′ - AACCCCATTCAGCGTCA -3′
*VCAM1*	Forward – 5′ - CCGGATTGCTGCTCAGATTGGA -3′ Reverse – 5′ - AGCGTGGAATTGGTCCCCTCA -3′
*IL6*	Forward – 5′ - GGTACATCCTCGACGGCATCT -3′ Reverse – 5′ - GTGCCTCTTTGCTGCTTTCAC -3′
*GAPDH*	Forward – 5′ – GCCAAAAGGGTCATCATCTC – 3′ Reverse – 5′ – GGCCATCCACAGTCTTCT – 3′
*HOTAIR*	Forward - 5′-CAGTGGGGAACTCTGACTCG-3′ Reverse – 5′-GTGCCTGGTGCTCTCTTACC-3′
*HULC*	Forward – 5′-TCATGATGGAATTGGAGCCTT-3′ Reverse – 5′-CTCTTCCTGGCTTGCAGATTG-3′
*MEG3*	Forward – 5′-GCCAAGCTTCTTGAAAGGCC-3′ Reverse – 5′-TTCCACGGAGTAGAGCGAGTC-3′
*NEAT1*	Forward – 5′-TGGCTAGCTCAGGGCTTCAG-3′ Reverse – 5′-TCTCCTTGCCAAGCTTCCTTC-3′
*UCA1*	Forward – 5′-CATGCTTGACACTTGGTGCC-3′ Reverse – 5′-GGTCGCAGGTGGATCTCTTC-3′
*MALAT-1*	Forward – 5′-TAGGAAGACAGCAGCAGACAGG-3′ Reverse – 5′-TTGCTCGCTTGCTCCTCAGT-3′

*ICAM1*, *VCAM1*, and *IL6* expression levels were calculated by the 2-ΔΔCt method. *GAPDH* was used as an endogenous control for data normalization. The data was also re-analyzed with 18s rRNA and did not show any difference as when *GAPDH* was used (*data not shown*). Relative enrichment of lncRNAs in RIP analysis was performed based on normalization to enrichment with just IgG.

### RNA Interference

The following siRNAs from Thermo Fisher Scientific were used against MALAT-1 – assay IDs n272231 and n272232; HULC – assay IDs n272667 and n272668; and UCA1 – assay IDs n272525 and n272526. HMECs were transiently transfected with both siRNAs targeting each lncRNA at the same time using Lipofectamine LTX (Thermo Fisher Scientific; 100 nM final concentration). Seventy-two hours post-transfection, cells were either left untreated or treated with 1 μg/ml LPS for 24 h before cells were harvested. Successful knockdown was verified by qRT-PCR.

### Animal Studies

Eleven-week C57BL/6 mice were intraperitoneally injected with a sub-lethal dose of LPS (1 mg/kg body mass). Control scramble siRNA or siRNA targeting HULC and UCA1 (13 μg/week in 100 μl volume) were administered by tail-vein injection, using equal volume mixtures of Lipofectamine^TM^ 2000. Two weeks later, mice were euthanized by CO_2_ narcosis. The aortic were carefully excised and fixed with 10% formalin solution. Paraffin sections (5 μm thickness) of aorta were prepared for immunohistochemistry (IHC) staining using anti-ICAM1 antibody (clone 1A29, Thermo Fisher Scientific), anti-VCAM1 (clone 429, Thermo Fisher Scientific), and anti-IL6 antibody (ab6672, Abcam). Images were scored based on percent staining by a pathologist blinded to the identity of the tissue specimens.

### Statistical Analysis

Data is represented as mean ± standard deviation (SD). Statistical significance was determined by the two-tailed Student’s *t*-test. A *P* < 0.05 was considered as statistically significant.

## Results

**FIGURE 1:**
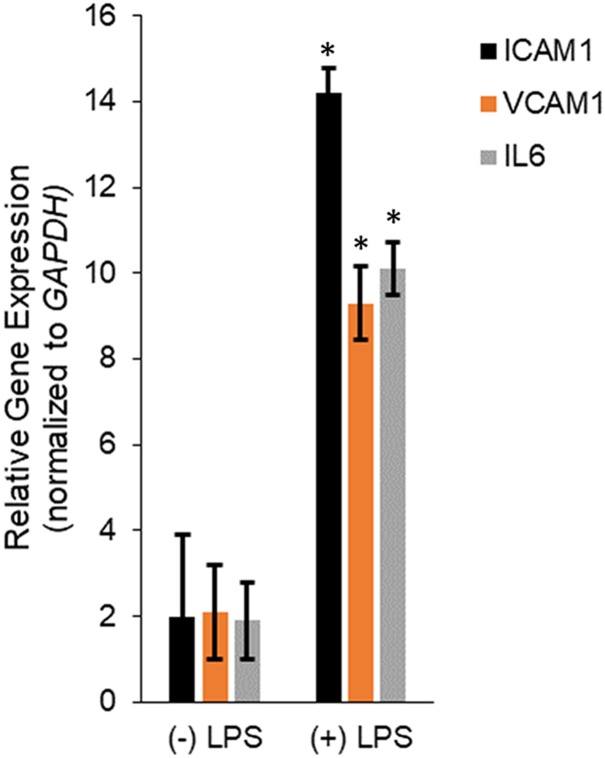
Transcription of *IL6*, *ICAM1*, and *VCAM1* encoding interleukin-6, intercellular adhesion molecule-1, and vascular cell adhesion molecule-1, respectively, were upregulated during LPS induced sepsis. Expression of *ICAM1*, *VCAM1*, and *IL6* mRNAs were determined by qRT-PCR in untreated or LPS-treated HMECs. Data represent mean ± standard deviation of three independent experiments, post-normalization to *GAPDH* expression. ^∗^*P* < 0.05, compared to untreated cells.

Human dermal microvascular endothelial cells were treated with LPS for 24 h and then RNA isolated from untreated and LPS treated HMECs were assayed for relative expression of the pro-inflammatory mediators, *ICAM1*, *VCAM1*, and *IL6*. LPS treatment significantly induced *ICAM1* (14.2 ± 0.57 in LPS vs. 2 ± 1.9 in untreated), *VCAM1* (9.3 ± 0.87 in LPS vs. 2.1 ± 1.09 in untreated), and *IL6* (10.1 ± 0.61 in LPS vs. 1.9 ± 0.9 in untreated; *P* < 0.05 in each case; Figure 1), confirming successful induction of *in vitro* sepsis.

**FIGURE 2:**
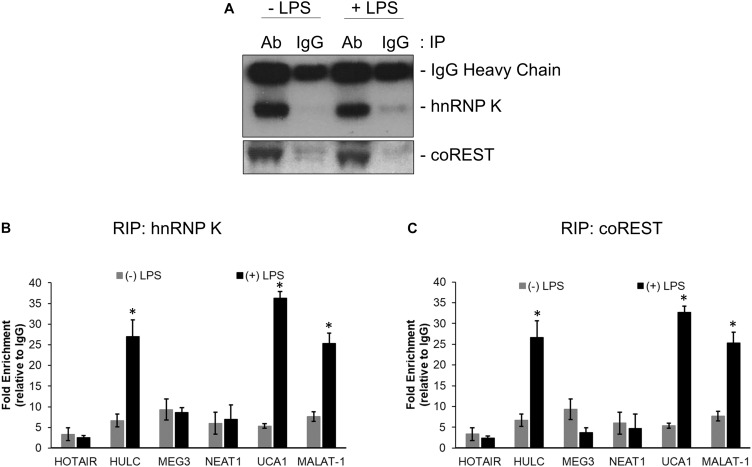
HULC, UCA1, and MALAT-1 lncRNAs were upregulated following LPS induced sepsis in HMECs. Nuclear extracts prepared from untreated or LPS treated HMECs were subjected to immunoprecipitation using anti-hnNRP K and anti-coREST **(A)** antibodies. RNA was isolated from immunoprecipitates and subjected to quantitative RT-PCR to detect relative enrichment of indicated lncRNAs in anti-hnRNP K immunoprecipitate **(B)** and anti-coREST immunoprecipitate **(C)**, compared to IgG immunoprecipitates. Data represent relative enrichment compared to IgG precipitates. ^∗^*P* < 0.05 compared to untreated cells.

RNA immunoprecipitation was performed using anti-hnRNP K, anti-coREST, and the corresponding IgG antibodies from lysates obtained from untreated and LPS treated HMEC cells. Immunoprecipitation in each case was confirmed by immunoblotting (Figure 2A). Relative enrichment, compared to IgG, for HOTAIR, HULC, MEG3, NEAT1, UCA1, and MALAT-1 lncRNAs were determined in hnRNP K (Figure 2B) and co-REST (Figure 2C) immunoprecipitates-derived RNA. HULC, UCA1, and MALAT-1 were significantly enriched in both RIP profiles (*P* < 0.05 in each case compared to RIP from lysates obtained from untreated cells). There was no significant difference in enrichment obtained in the LPS treated lysates with hnRNP K or coREST antibodies, indicating equivalent affinity for the lncRNAs for the CMP components.

**FIGURE 3:**
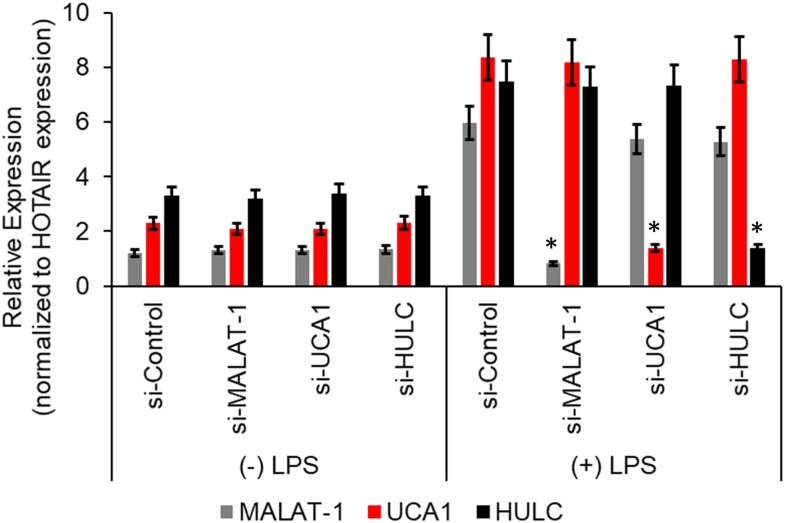
Relative expression of HULC, UCA1, and MALAT-1 expression in ± LPS treated HMECs post-transfection with siRNAs targeting HULC, UCA1, and MALAT-1 or a scrambled control were performed by qRT-PCR. No difference in expression was noted in MALAT-1 (gray bars), UCA1 (red bars), or HULC (black bars) in the (–) LPS group following transfection of the siRNAs. In the (+) LPS group transfected with control scrambled siRNA expression was significantly higher for all three lncRNAs. Successful knockdown of the individual lncRNA following transfection of siRNA targeting that lncRNA was verified compared to the si-Control in (+) LPS group. Data represent mean ± standard deviation of three independent experiments, post-normalization to HOTAIR expression. ^∗^*P* < 0.05, compared to control scramble siRNA transfected LPS treated HMECs.

We next wanted to determine if there was a correlation between induction of the proinflammatory markers and increase in lncRNA enrichment in the CMP complex, we transiently transfected the HMECs with either a control scramble siRNA or two independent siRNAs targeting either MALAT-1, UCA1, or HULC. Seventy-two hours post-transfection, the HMECs were either left untreated or treated with LPS for an additional 24 h. Quantitative RT-PCR was performed to confirm successful silencing of the lncRNA expression by the respective siRNAs. Compared to the control scramble siRNA, the siRNAs specific to MALAT-1, UCA1, or HULC, respectively, significantly down regulated the expression of MALAT-1, UCA1, and HULC in LPS-treated HMECs (Figure 3).

**FIGURE 4:**
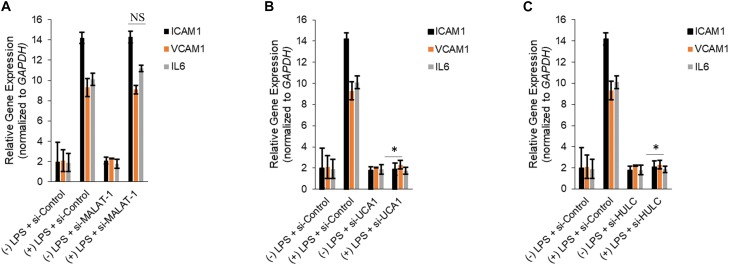
Downregulation of UCA1 and HULC, but not MALAT-1, using siRNAs inhibits sepsis associated induction of *ICAM1*, *VCAM1*, and *IL6*. Expression of *ICAM1*, *VCAM1*, and *IL6* mRNAs were determined by qRT-PCR in HMECs, either mock transfected or transfected with siRNAs targeting MALAT-1 **(A)**, UCA1 **(B)**, and HULC **(C)**. Data represent mean ± standard deviation of three independent experiments, post-normalization to *GAPDH* expression. ^∗^*P* < 0.05, compared to LPS treated HMECs with no siRNA transfection.

**FIGURE 5:**
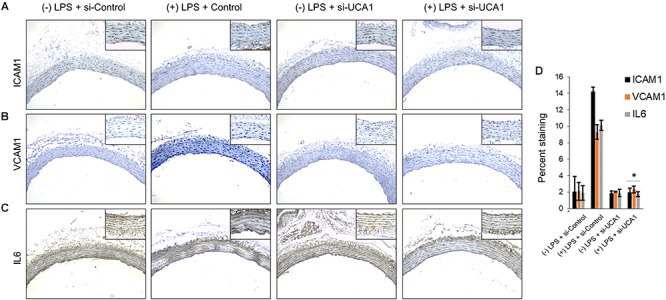
Downregulation of UCA1 using siRNA inhibits sepsis associated induction of ICAM1, VCAM1, and IL6 *in vivo*. The aorta was isolated and the expression levels of ICAM1, VCAM1, and IL-6 were detected by immunohistochemical staining with IHC. Shown are representative images obtained at 100× magnification (inset 400× magnification) of aortic tissue in indicated experimental groups stained with antibody against **(A)** ICAM1, **(B)** VCAM1, and IL6 **(C)**. **(D)** Quantification of images obtained from 6 animals of each experimental group. ^∗^*P* < 0.05, compared to corresponding specimen in +LPS + siRNA-scramble control group.

**FIGURE 6:**
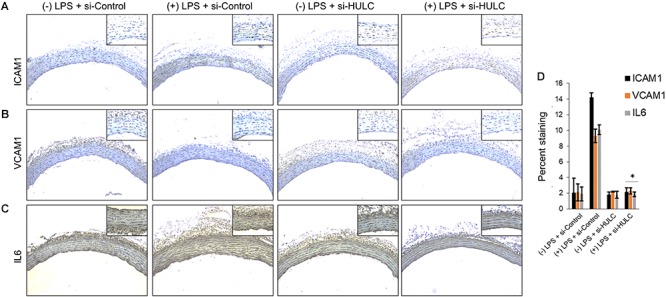
Downregulation of HULC using siRNA inhibits sepsis associated induction of ICAM1, VCAM1, and IL6 *in vivo*. The aorta was isolated and the expression levels of ICAM1, VCAM1, and IL-6 were detected by immunohistochemical staining with IHC. Shown are representative images obtained at 100× magnification (inset 400× magnification) of aortic tissue in indicated experimental groups stained with antibody against **(A)** ICAM1, **(B)** VCAM1, and IL6 **(C)**. **(D)** Quantification of images obtained from 6 animals of each experimental group. ^∗^*P* < 0.05, compared to corresponding specimen in +LPS + siRNA-scramble control group.

Silencing of MALAT-1 did not result in significant decrease in expression of *ICAM1*, *VCAM1*, and *IL6* in the LPS treated HMECs (*ICAM1* 14.2 ± 1.9 folds in LPS group vs. 14.3 ± 0.58 in LPS + si-MALAT-1 group, *P* > 0.05; *VCAM1*: 9.3 ± 1.09 folds in LPS group vs. 9.1 ± 0.41 in LPS + si-MALAT-1 group, *P* > 0.05; and, *IL6*: 10.1 ± 0.9 folds in LPS group vs. 11.2 ± 0.31 in LPS + si-MALAT-1 group, *P* > 0.05) (Figure 4A). Silencing of UCA1 significantly decreased the expression of *ICAM1*, *VCAM1*, and *IL6* in the LPS treated HMECs to those observed in the endogenous untreated HMECs (*ICAM1* 14.2 ± 1.9 folds in LPS group vs. 1.9 ± 0.98 in LPS + si-UCA1 group, *P* < 0.05; *VCAM1*: 9.3 ± 1.09 folds in LPS group vs. 2.31 ± 0.83 in LPS + si-UCA1 group, *P* < 0.05; and, *IL6*: 10.1 ± 0.9 folds in LPS group vs. 1.75 ± 0.83 in LPS + si-UCA1 group, *P* < 0.05) (Figure 4B, 5). Silencing of HULC lncRNA also had a similar effect to silencing of UCA1 lncRNA. Silencing of HULC significantly decreased the expression of *ICAM1*, *VCAM1*, and *IL6* in the LPS treated HMECs to those observed in the endogenous untreated HMECs (*ICAM1* 14.2 ± 1.9 folds in LPS group vs. 2.1 ± 0.04 in LPS + si-HULC group, *P* < 0.05; *VCAM1*: 9.3 ± 1.09 folds in LPS group vs. 2.3 ± 0.02 in LPS + si-HULC group, *P* < 0.05; and, *IL6*: 10.1 ± 0.9 folds in LPS group vs. 1.86 ± 0.07 in LPS + si-HULC group, *P* < 0.05) (Figure 4C, 6). Taken together, these results indicate that increase in proinflammatory *ICAM1*, *VCAM1*, and *IL6* in HMECs during LPS induced sepsis *in vitro* is at least in part due to increase in expression of the lncRNAs HULC and UCA1.

## Discussion

It has been shown comprehensively that lncRNAs regulate gene expression during development, normal physiological processes and in aberrant disease contexts (Perez et al., 2008; Guttman et al., 2011). Even though lncRNAs do not encode protein products changes in their levels of expression seem to regulate expression of messenger RNA targets in these scenarios.

The results from the current study show that along with lnc-IL17R (Chowdhury et al., 2017), lncRNAs MALAT-1, HULC, and UCA1 are also upregulated during LPS induced sepsis. Among the upregulated lncRNAs, HULC and UCA1 seem to regulate induction in expression of the messenger RNAs for the proinflammatory interleukin-6 (IL-6), intercellular adhesion molecule-1 (ICAM-1), and vascular cell adhesion molecule-1 (VCAM-1). Given our *in vivo* results it seems that targeted knockdown of HULC and UCA1 might be a viable therapeutic option for sepsis, even though long-term stability and directed organ-specific delivery will be potential problems.

It remains to be determined how, if at all, upregulation of MALAT-1 impinges on LPS induced sepsis. It is highly possible that there are other targets whose expression is also modulated following induction in expression of the HULC, UCA1, and MALAT-1 lncRNAs during sepsis in endothelial cells. This becomes even more important because it has been shown that MALAT-1 actually mediates inflammation in traumatic brain injury. It also remains to be determined if the identity of the lncRNAs and their gene targets remain same across different forms of sepsis, and during LPS induced sepsis *in vivo*. The CRNDE lncRNA has been shown to trigger inflammation through the TLR3-NF-κB cytokine signaling pathway. Whether CRNDE is involved in sepsis remains to be determined.

The current study does suffer from some technical limitations. Foremost is RIP was performed using two of the four components of the CMP complex. Whether similar enrichment of lncRNAs are observed when RIP is performed using Sin3A and polycomb repressor complex 2 is imperative to determine as affinity of the lncRNAs might not be the same for all four components of the CMP. Additionally, it remains to be determined if other lncRNAs are also involved in the pathogenesis of sepsis.

In addition, RNAseq or lncRNA microarray should be performed on the RIP immunoprecipitates to identify the entire cohort of differentially enriched lncRNAs. Finally, we evaluated lncRNA enrichment in the CMP complex at one dose and one time point post-LPS treatment. It remains to be determined if the lncRNAs’ enrichment within the CMP complex is time and dose dependent following treatment with LPS.

## Ethics Statement

The study was approved by IACUC of The First Hospital of Jilin University.

## Author Contributions

NL designed the experiments. YC, YF, and Y-fS performed the experiments and analyzed the data. YC and NL prepared the manuscript. All authors have read the manuscript.

## Conflict of Interest Statement

The authors declare that the research was conducted in the absence of any commercial or financial relationships that could be construed as a potential conflict of interest.
